# Beyond Weight Loss: Skeletal Muscle Health During Incretin-Based Therapy in Patients with Diabesity

**DOI:** 10.3390/nu18162654

**Published:** 2026-08-14

**Authors:** Edoardo Luigi Maria Mollero, Ivan Dozzani, Emilia Biamonte, Giulia Bendotti, Paolo Marzullo, Gianluca Aimaretti, Marco Gallo

**Affiliations:** 1 Endocrinology and Metabolic Diseases Unit, Department of Internal Medicine and Emergency Medicine, IRCCS AOU “SS Antonio e Biagio e Cesare Arrigo”, Via Venezia, 16, 15121 Alessandria, Italy; emilia.biamonte@ospedale.al.it (E.B.); giulia.bendotti@ospedale.al.it (G.B.); 2 Department of Statistics, Computer Science, Applications “Giuseppe Parenti”, University of Florence, Viale Giovanni Battista Morgagni 59, 50134 Firenze, Italy; 3 Independent Researcher, 50142 Firenze, Italy; ivandozzani@gmail.com; 4 Division of Endocrinology, Department of Translational Medicine, University of Eastern Piedmont, Via Solaroli 17, 28100 Novara, Italy; paolo.marzullo@med.uniupo.it; 5 Division of Endocrinology and Metabolism, Department of Medical Sciences, University of Turin, Corso Dogliotti 14, 10126 Torino, Italy; gianluca.aimaretti@unito.it

**Keywords:** GLP-1 receptor agonists, tirzepatide, semaglutide, skeletal muscle, muscle quality, sarcopenia, obesity, type 2 diabetes, nutrition, resistance training

## Abstract

**Background:** Incretin-based therapies (IBTs), including glucagon-like peptide-1 receptor agonists (GLP-1 RAs) and dual glucose-dependent insulinotropic polypeptide/glucagon-like peptide-1 receptor agonists (GIP/GLP-1 RAs), have transformed the management of obesity and type 2 diabetes mellitus (T2DM). Although these agents provide substantial metabolic and cardiometabolic benefits, their effects on skeletal muscle, particularly in older adults at increased risk of sarcopenia and functional decline, remain incompletely understood. **Methods:** This narrative review synthesizes evidence from randomized controlled trials (RCTs) and observational studies evaluating the effects of semaglutide and tirzepatide on skeletal muscle mass (SMM), muscle quality, strength, and physical performance. Current evidence on nutritional and exercise strategies aimed at preserving skeletal muscle during IBT was also reviewed. **Results:** Both semaglutide and tirzepatide induce substantial weight loss accompanied by reductions in lean body mass (LBM). However, current evidence indicates that LBM loss is generally proportional to the magnitude of weight loss and should not be interpreted as a direct surrogate for skeletal muscle loss or drug-induced myotoxicity. Tirzepatide appears to improve skeletal muscle composition by reducing muscle fat infiltration (MFI), whereas semaglutide shows more heterogeneous effects on muscle strength and physical performance, particularly in older or frail individuals. Emerging evidence highlights the importance of muscle quality, nutritional adequacy, and resistance exercise as key determinants of muscle preservation, although functional outcomes and data in older adults remain limited. **Conclusions:** The effects of incretin-based therapies (IBTs) on skeletal muscle are multifactorial and influenced by age, baseline muscle reserve, nutritional status, and physical activity. Preserving skeletal muscle health should be considered an integral component of obesity management through individualized nutritional care, adequate protein intake, resistance exercise, and regular functional assessment. Future research should prioritize the standardized evaluation of muscle quality and function and determine whether targeted nutritional interventions can improve the quality of weight loss and support healthy aging during IBT.

## 1. Introduction

The population of the Western world is progressively aging and is increasingly burdened by chronic conditions with rising prevalence [1,2]. Among these, partly due to sedentary lifestyles and the central role of food in daily life, type 2 diabetes (T2DM) and obesity have reached epidemic proportions, leading to the coining of the term “diabesity,” with its associated impact of comorbidities and complications [3,4].

In recent years, the development of novel glucagon-like peptide-1 receptor agonists (GLP-1 RAs) and dual GLP-1/glucose-dependent insulinotropic polypeptide receptor agonists (GLP-1/GIP RAs), particularly those administered as once-weekly subcutaneous injections, has significantly improved glycemic control and weight management in these patients, while also conferring benefits to cardiovascular, renal, and overall metabolic outcomes, as extensively documented in randomized controlled trials (RCTs) [5,6] and reflected in international guidelines [7].

However, an aspect that remains insufficiently explored concerns the impact of these therapies on skeletal muscle, particularly in older individuals, who are already at an increased risk of sarcopenia and functional decline [8,9].

The aim of this narrative review is, after a brief overview of the definition and clinical-diagnostic framework of sarcopenia, to summarize the current evidence on the effects of semaglutide and tirzepatide on skeletal muscle and to discuss potential strategies to mitigate drug-associated muscle loss or weakness, with a focus on nutritional interventions and physical activity.

## 2. Materials and Methods

A narrative literature review was conducted through a comprehensive search of the PubMed and Embase databases, including studies published up to June 2026. Search terms included combinations of: “GLP-1 receptor agonists”, “GLP-1/GIP receptor agonists”, “semaglutide”, “tirzepatide”, “skeletal muscle”, “lean mass”, “body composition”, “muscle strength”, “muscle quality”, “sarcopenia”, “nutrition”, and “exercise”.

Initially, the search strategy targeted studies conducted in older adults (≥65 years). However, due to limited data in this population, the inclusion criteria were expanded to studies in adult populations when relevant outcomes related to skeletal muscle (e.g., lean mass, muscle strength, physical performance, or muscle quality) were reported.

Eligible studies included RCTs, observational studies, mechanistic investigations, and relevant international guidelines. Particular emphasis was placed on studies assessing body composition using validated techniques such as dual-energy X-ray absorptiometry (DXA), bioelectrical impedance analysis (BIA), and magnetic resonance imaging (MRI), as well as those reporting functional outcomes (e.g., handgrip strength, gait speed, and physical performance measures).

Titles and abstracts were screened, and full texts were assessed when relevant. Only articles published in English were included. Studies not reporting outcomes related to skeletal muscle or its functional correlates were excluded. Reference lists of selected articles were manually searched to identify additional relevant studies. Given the narrative design, a formal systematic review protocol (e.g., PRISMA) was not applied. However, efforts were made to include the most relevant and recent evidence. Given the heterogeneity of study designs and outcome measures, Table 1 summarizes the main clinical and methodological characteristics of the available evidence on incretin-based therapies (IBTs) and skeletal muscle outcomes.

## 3. Results

### 3.1. Sarcopenia: Operational Definition and Clinical Framework

Sarcopenia is a progressive and generalized skeletal muscle disorder characterized by an age-related decline in muscle mass, strength, and physical performance, leading to an increased risk of frailty, falls, disability, and mortality [8,22,23]. It is particularly relevant in older adults and in individuals with chronic metabolic diseases such as T2DM and obesity, in whom metabolic inflammation, insulin resistance, and physical inactivity may accelerate muscle deterioration.

The current operational definition is based on the revised European consensus from the European Working Group on Sarcopenia in Older People (EWGSOP2), which identifies low muscle strength as the primary parameter of sarcopenia, supported by low muscle quantity or quality for diagnosis confirmation and low physical performance for severity classification [8].

Complementary frameworks, including the Sarcopenia Definition and Outcomes Consortium (SDOC), emphasize muscle strength and physical function as the strongest predictors of adverse outcomes, with reduced emphasis on muscle mass as an isolated criterion [22]. Similarly, the Foundation for the National Institutes of Health (FNIH) Sarcopenia Project provides data-driven cutoffs for appendicular lean mass, grip strength, and gait speed, supporting the identification of clinically relevant phenotypes associated with mobility limitations and disability risk [23].

Muscle strength is most commonly assessed using handgrip dynamometry, while physical performance is evaluated using gait speed, the Short Physical Performance Battery (SPPB), or chair-rise tests. Muscle quantity or mass is typically measured using DXA, BIA, or cross-sectional imaging techniques such as Computed Tomography (CT) and MRI, which also allow the evaluation of muscle quality, including muscle fat infiltration (MFI) and intermuscular adipose tissue [22,23]. It is important to distinguish between lean body mass (LBM), fat-free mass (FFM) and skeletal muscle mass (SMM), which are frequently used interchangeably; however, they represent distinct body composition concepts. Fat mass (FM) refers exclusively to adipose tissue, whereas FFM includes all non-fat components of the body, including skeletal muscle, bone mineral content, organs, connective tissues, and body water. LBM is often used operationally as a surrogate for FFM in body composition studies, although minor methodological differences exist between these terms. SMM represents only one component of FFM and specifically refers to contractile muscle tissue. DXA and BIA primarily estimate LBM, whereas CT and MRI provide a more direct assessment of skeletal muscle volume and composition. Consequently, reductions in DXA-derived LBM should not be interpreted as equivalent to proportional losses of contractile skeletal muscle. Most clinical trials report DXA-derived LBM rather than direct measurements of skeletal muscle [24,25,26].

Sarcopenic obesity represents a clinically relevant phenotype characterized by the coexistence of excess adiposity and impaired muscle function and is associated with higher risks of disability and metabolic complications compared with either condition alone [24,25,26].

Overall, these consensus definitions highlight sarcopenia as a multidimensional syndrome in which muscle strength and physical performance are central determinants of clinical outcomes, while muscle mass and quality provide complementary diagnostic information. This framework is particularly relevant when evaluating pharmacologically induced weight loss and its effects on skeletal muscle [8,9,22,23].

### 3.2. Effects of Semaglutide on Skeletal Muscle

Semaglutide is a GLP-1 RA that induces weight loss primarily through appetite suppression, delayed gastric emptying, and improved glucose homeostasis [27,28]. Beyond its metabolic effects, it also exerts anti-inflammatory and cardioprotective actions [29,30,31].

Within the GLP-1 RA literature, some degree of LBM or FFM loss is expected during weight reduction; however, the magnitude of this loss and its functional consequences may be particularly relevant in vulnerable populations such as older adults.

Although skeletal muscle expresses relatively low levels of the GLP-1 receptor, indirect effects mediated by improvements in insulin sensitivity, substrate utilization, and overall energy balance are considered biologically meaningful [32].

Although oral semaglutide has demonstrated efficacy in improving glycemic control and promoting weight loss, evidence specifically addressing its effects on skeletal muscle remains limited. Available data suggest reductions in visceral adipose tissue alongside preservation of LBM and SMM, potentially improving the skeletal muscle-to-visceral fat ratio [10]. Nevertheless, the most robust evidence on SMM outcomes is currently derived from studies using subcutaneous semaglutide. Notably, the lack of control groups, functional endpoints, and high-precision imaging techniques limits the ability of existing studies to exclude subtle or long-term effects on SMM, particularly in older populations [10,33].

Several prospective and observational studies provide more direct evidence on changes in LBM and muscle function during subcutaneous semaglutide treatment. The SEMALEAN prospective study evaluated semaglutide 2.4 mg in individuals with obesity using DXA, handgrip strength, and resting energy expenditure at baseline, 7 months, and 12 months. This real-world longitudinal cohort included patients with severe obesity and provides one of the most detailed combined structural and functional assessments currently available. The study reported significant weight loss, primarily driven by reductions in FM, while LBM showed an initial decline followed by stabilization over time. Substantially, appendicular SMM tracked in parallel with total LBM, suggesting no disproportionate depletion of muscle compartments.

Crucially, SEMALEAN provides robust functional data: handgrip strength increased over time, and the prevalence of sarcopenic obesity decreased, indicating that reductions in LBM did not necessarily translate into impaired muscle function. In addition, resting energy expenditure normalized to LBM increased during follow-up, suggesting improved metabolic efficiency. Subgroup analyses highlighted heterogeneity according to clinical characteristics, reinforcing the importance of context when interpreting body composition changes [11].

More recently, additional evidence from older populations has highlighted the need for caution when interpreting body composition changes during semaglutide therapy. In a 24-month retrospective cohort study of older adults with T2DM, Ren et al. reported greater declines in appendicular SMM, muscle strength, and physical performance among semaglutide-treated individuals compared with controls. A higher semaglutide dose, lower baseline appendicular skeletal muscle index, and poorer baseline gait speed were identified as independent predictors of muscle loss, suggesting that frail older adults may require closer monitoring during treatment. Although the retrospective design precludes causal inference, these findings reinforce the importance of individualized assessment in vulnerable populations [12].

Within the STEP program, DXA sub-analyses have shown reductions in LBM among semaglutide-treated participants compared with placebo, although these findings are largely derived from younger or middle-aged populations [32]. More recently, the STEP UP phase 3b trial evaluating once-weekly semaglutide 7.2 mg extends this evidence by demonstrating a clear dose–response relationship in weight reduction, with mean weight loss approaching ~20% at 72 weeks, greater than that observed with the 2.4 mg dose and markedly superior to placebo. Available data suggest that these larger weight reductions are primarily driven by FM loss, with a proportional change in LBM that does not indicate a selective detrimental effect on skeletal muscle [34].

Consistent with these findings, additional randomized evidence from T2DM populations supports a broadly similar pattern. In the SUSTAIN 8 DXA substudy (n = 178), semaglutide was compared with canagliflozin over 52 weeks, showing reductions in both FM and LBM [13]. Importantly, absolute and relative LBM should be distinguished when interpreting these findings. Absolute LBM generally decreases during weight loss, whereas its relative proportion may increase because FM declines to a greater extent. Therefore, an increase in relative LBM should not be interpreted as evidence of skeletal muscle gain but rather as a reflection of improved overall body composition [25,26].

Furthermore, findings become more heterogeneous in older populations and in individuals with T2DM. A pilot study in older obese patients with T2DM showed that semaglutide significantly reduced FM and body fat percentage after 3 months, while preserving the relative proportion of SMM and regional muscle distribution across limbs, suggesting the preservation of muscle mass relative to body weight despite weight loss [14].

In accordance with this concern, Qaisar et al. reported significant declines in muscle strength, physical performance, and muscle mass indices in older men with T2DM treated with semaglutide, alongside increases in biomarkers of neuromuscular impairment [15].

Additional real-world evidence supports these observations. A 26-week prospective study in adults with T2DM and obesity showed modest changes in handgrip strength despite reductions in LBM [35]. Similarly, bioimpedance-based analyses demonstrated significant reductions in LBM despite metabolic improvements [16].

Imaging-based studies provide further structural insights. In the SLIM LIVER study, semaglutide treatment was associated with a reduced psoas muscle volume without significant deterioration in physical performance [17].

Finally, studies in specific comorbidity settings further highlight the complexity of interpretation. In patients with T2DM and chronic kidney disease (CKD), semaglutide was associated with reductions in absolute muscle mass alongside changes in relative indices, emphasizing the importance of how body composition outcomes are expressed [36].

Importantly, prospective evidence specifically evaluating the effects of GLP-1 RAs on skeletal muscle health and physical function in older adults remains scarce. To address this knowledge gap, Cortes et al. recently designed an open-label RCT investigating the effects of semaglutide in older adults with overweight or obesity and insulin resistance. Participants will receive semaglutide in combination with a lifestyle intervention or lifestyle intervention alone, with follow-up including comprehensive assessments of body composition, muscle strength, physical performance, and biological markers of aging. Muscle function will be evaluated through standardized measures such as handgrip strength, gait speed, chair stand performance, and the SPPB, while body composition will be assessed using DXA. The study will also investigate circulating biomarkers related to inflammation, cellular senescence, and metabolic health, providing mechanistic insights into the interaction between semaglutide, aging, and skeletal muscle. Although the results are not yet available, this trial is expected to provide valuable prospective evidence on whether semaglutide-induced weight loss is accompanied by the preservation, or even improvement, of muscle function and overall physical performance, helping to clarify the balance between reductions in LBM and clinically meaningful functional outcomes in older adults [18].

### 3.3. Effects of Tirzepatide on Muscle and Body Composition

The introduction of tirzepatide, a first-in-class GLP-1/GIP RA, has fundamentally reshaped the therapeutic landscape of obesity and T2DM [6,37,38]. However, the substantial body weight reduction achieved with this agent raises clinically relevant concerns regarding the quality of weight loss, particularly in older adults. In this population, the preservation of SMM and function extends far beyond simple body composition metrics, representing a cornerstone for the prevention of frailty, falls, disability, and progressive functional decline [19,38,39].

Data from the SURMOUNT clinical development program provide a robust framework for understanding tirzepatide-induced body composition changes [6,40]. In the DXA substudy of SURMOUNT-1, Look et al. demonstrated that treatment with tirzepatide 15 mg produced a mean body weight reduction of 21.4% at week 72. Importantly, this reduction was predominantly driven by FM loss. Specifically, FM decreased by an estimated treatment difference of 25.7% versus placebo, whereas the corresponding reduction in LBM was 8.3% [40]. Overall, approximately 74% of total weight loss was attributable to FM reduction and 26% to LBM loss, a proportion closely aligned with the classical 3:1 ratio reported during lifestyle-induced weight loss and bariatric surgery [40,41,42].

A particularly reassuring finding was that these proportions remained broadly consistent across the age and sex subgroups evaluated in SURMOUNT-1, including participants aged ≥65 years [40]. This suggests that, within the age range represented in the trial, older adults did not experience a disproportionate loss of LBM compared with younger participants, consistent with previous observations from lifestyle-induced weight loss studies [40,41]. Nevertheless, because adults aged ≥75 years were only sparsely represented, these findings should not be extrapolated to the oldest old without caution. Despite the absolute decline in LBM, the substantially greater depletion of adipose tissue resulted in a net improvement in body composition profile, which is particularly relevant in the context of sarcopenic obesity [39].

Beyond the quantitative DXA data, MRI offers a more granular characterization of skeletal muscle quality and ectopic fat distribution. The SURPASS-3 MRI substudy evaluated adults with T2DM inadequately controlled on metformin with or without Sodium-Glucose Co-Transporter-2 inhibitors (SGLT2i), randomized to tirzepatide or insulin degludec. This post hoc analysis demonstrated that although total muscle volume and muscle volume Z-scores decreased during tirzepatide treatment, the magnitude of reduction was consistent with population-based estimates for a comparable degree of weight loss. In other words, the observed reduction in muscle volume did not exceed physiological expectations associated with substantial weight loss. The most clinically relevant finding of the MRI substudy, however, concerns MFI. Unlike muscle volume, which followed expected weight-loss trajectories, MFI decreased significantly more than predicted by population-based models derived from the UK Biobank [20]. This observation may be particularly relevant in older adults, in whom myosteatosis and intramuscular adipose tissue typically increase with aging and T2DM progression.

The subsequent commentary by Yen and Li further reinforced the significance of this finding, emphasizing that tirzepatide not only counteracted the natural age-related increase in MFI but actively promoted its reduction beyond expected physiological trends [43]. This qualitative remodeling of skeletal muscle may have clinical relevance; however, whether these imaging changes translate into meaningful improvements in muscle function or physical performance remains to be established.

Indeed, intramuscular lipid accumulation is strongly associated with reduced contractile efficiency, altered fiber pennation angle, increased connective tissue stiffness, impaired oxygen and nutrient delivery, and chronic local inflammation. By reducing ectopic lipid deposition within skeletal muscle, tirzepatide may improve muscle quality, metabolic flexibility, and local insulin sensitivity, potentially mitigating some of the functional risks associated with LBM reduction [43,44].

In this regard, a direct comparison with semaglutide is of particular interest. The SURPASS-2 trial established the superiority of tirzepatide over semaglutide in both glycemic control and overall body weight reduction [45]. Nonetheless, from a body composition standpoint, available evidence suggests that the proportion of LBM loss relative to total weight loss is broadly comparable between the two agents, generally ranging between 20% and 40% [5,6,19,45].

The key difference lies in the absolute magnitude of total weight loss: because tirzepatide typically induces greater weight reduction, the absolute loss of LBM in kilograms may be numerically higher. Nevertheless, the GIP receptor agonism component may confer additional metabolic advantages by directly modulating adipose tissue lipid buffering and ectopic fat partitioning [46]. This optimized nutrient distribution may facilitate a more favorable reduction in myosteatosis and intermuscular adipose tissue, which could be particularly advantageous in older adults with a limited muscle reserve and high risk of sarcopenic obesity.

These findings are further supported by the recent systematic review by Hidalgo Ramos and colleagues, which specifically evaluated the effects of tirzepatide on SMM in adults across RCTs. The review included available DXA and MRI-based substudies from the SURMOUNT-1 and SURPASS-3 clinical programs and suggested that tirzepatide-induced weight loss is predominantly characterized by FM reduction with the relative preservation of LBM. Moreover, MRI analyses indicated favorable changes in muscle composition, including reductions in MFI, supporting the hypothesis that tirzepatide may improve aspects of muscle quality despite modest reductions in absolute muscle volume. Within the populations evaluated, approximately 75% of weight loss was attributable to adipose tissue, whereas LBM reduction remained consistent with expected physiological weight-loss trajectories [21]. However, it is important to emphasize that the available evidence does not allow this body composition pattern to be generalized to adults aged ≥75 years. The commonly cited 3:1 ratio between FM and LBM loss, derived primarily from lifestyle-induced weight loss studies, has not been specifically validated in very old adults receiving tirzepatide. Furthermore, participants aged ≥75 years were underrepresented in the SURMOUNT and SURPASS clinical programs, limiting the generalizability of these findings to this population [19,20].

Across studies, body composition findings should be interpreted in light of the assessment method. Most RCTs report DXA-derived LBM, whereas MRI studies quantify skeletal muscle volume and MFI. Because these techniques assess different biological compartments, direct comparisons should be interpreted cautiously [24,25,26]. The integrated effects of semaglutide and tirzepatide on skeletal muscle are summarized in Table 2.

**Figure 1:**
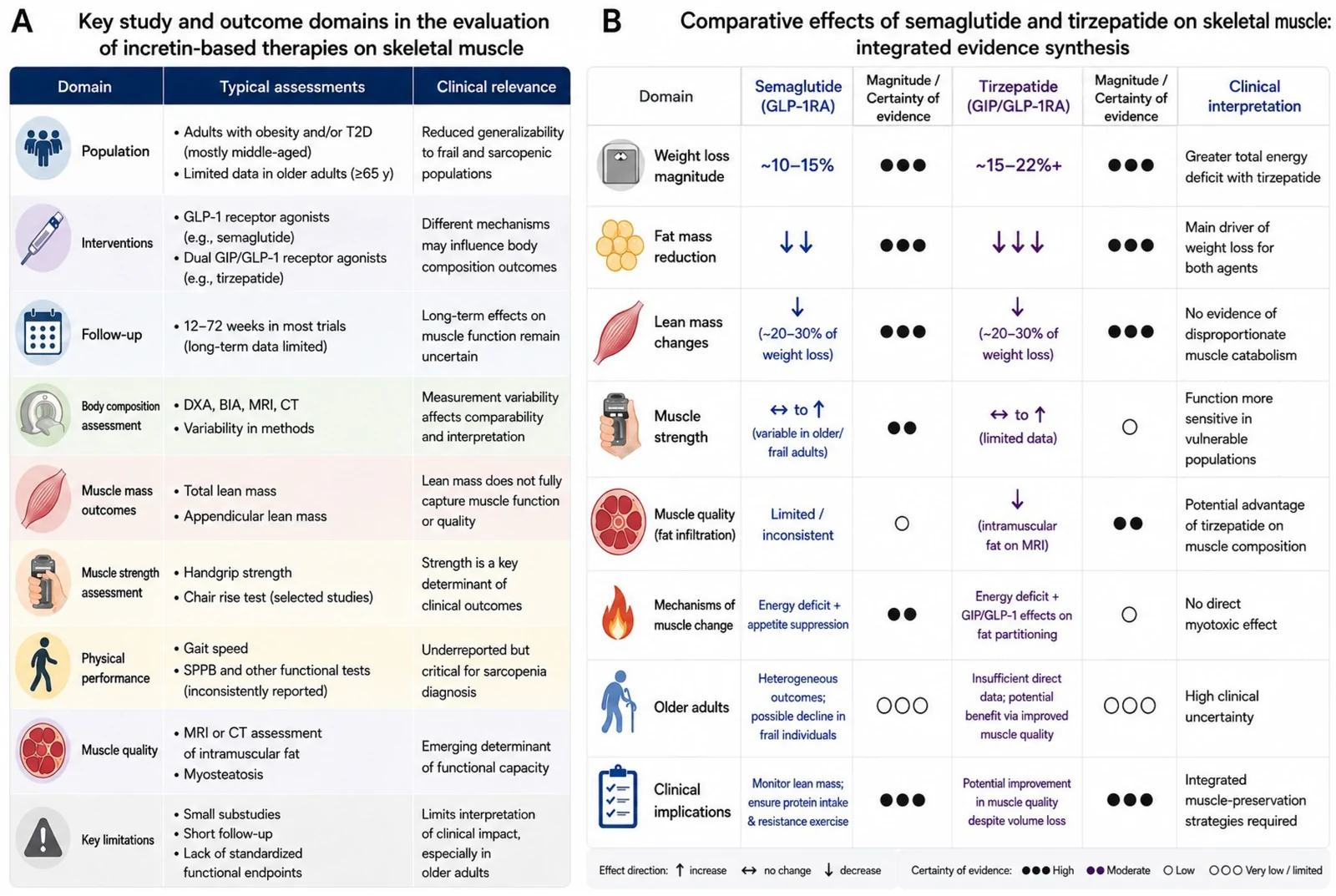
Effects of semaglutide and tirzepatide on skeletal muscle: integrated evidence synthesis; (**A**) key study and outcome domains in Incretin-Based Therapies (IBTs) on skeletal muscle; (**B**) graphical presentation of integrated evidence synthesis. **Abbreviations:** BIA, Bioelectrical Impedance Analysis; CT, Computed Tomography; DXA, Dual-Energy X-ray Absorptiometry; GIP, Glucose-Dependent Insulinotropic Polypeptide; GLP-1RA, Glucagon-Like Peptide-1 Receptor Agonist; MRI, Magnetic Resonance Imaging; RCTs, Randomized Controlled Trials; T2DM, Type 2 Diabetes Mellitus; IBTs, Incretin-Based Therapies.

To enhance visual interpretation of the available evidence, the key findings reported in Table 1 and Table 2 are summarized in the conceptual framework illustrated in Figure 1.

The quality of the evidence underlying each domain summarized in Figure 1B is presented separately in Table 3.

### 3.4. Strategies for Muscle Preservation

#### 3.4.1. Nutritional Strategies

The weight-reducing effects of IBTs are largely mediated by decreased appetite and energy intake. While this represents a key therapeutic advantage, it may also pose nutritional challenges, particularly in older adults, who are especially vulnerable to inadequate dietary intake. Reduced caloric consumption, when not carefully managed, may lead to insufficient protein intake and increase the risk of protein-energy malnutrition. This concern is further compounded by anabolic resistance, a hallmark of aging skeletal muscle characterized by a diminished anabolic response to dietary protein. When combined with reduced energy intake, this age-related impairment in muscle protein synthesis may accelerate LBM loss unless nutritional strategies are carefully optimized [47,48].

Ensuring adequate protein intake is therefore a cornerstone of muscle preservation during IBT. Current recommendations suggest a daily protein intake of at least 1.0–1.2 g/kg body weight in healthy older adults, with intakes of up to 1.5 g/kg/day recommended in the presence of acute or chronic disease or established sarcopenia [48,50]. However, achieving these targets may be challenging in patients receiving semaglutide or tirzepatide because of early satiety, delayed gastric emptying, and gastrointestinal adverse effects that may reduce spontaneous food intake.

Beyond total daily protein intake, attention should also be directed toward protein quality, meal distribution, and timing. High-quality protein sources rich in essential amino acids, particularly leucine, should be preferentially consumed and distributed evenly across three to four meals per day. Whenever feasible, each meal should provide approximately 25–30 g of high-quality protein, corresponding to approximately 2.5–3 g of leucine, in order to maximize postprandial muscle protein synthesis and partially overcome anabolic resistance in older adults [47,48]. Practical dietary strategies, including smaller and more frequent meals, texture modification, individualized meal timing, and the prioritization of protein-rich foods before other meal components, may facilitate adequate nutrient intake while improving treatment tolerability [50].

In addition to protein quantity and distribution, specific nutritional components have been investigated as potential strategies to counteract age-related anabolic resistance and support skeletal muscle preservation during periods of energy restriction. β-hydroxy-β-methylbutyrate (HMB), a metabolite derived from leucine, has demonstrated anti-catabolic properties and potential benefits for muscle mass and physical function, particularly in older adults with inadequate protein intake, acute illness, or an increased risk of muscle loss [51]. Although direct evidence in individuals receiving IBTs is currently unavailable, HMB supplementation may represent a potential adjunctive strategy in selected patients experiencing substantial weight loss, reduced dietary intake, or increased vulnerability to muscle decline.

Omega-3 (ω-3) polyunsaturated fatty acids (PUFAs) have also been proposed as potential modulators of skeletal muscle health through their anti-inflammatory effects and their ability to enhance anabolic sensitivity to dietary protein and resistance exercise. Evidence from studies in older adults suggests that ω-3 supplementation may improve muscle protein synthesis responses and may contribute to the preservation of muscle function; however, the clinical relevance of these findings during incretin-induced weight reduction remains to be established [52]. Similarly, leucine-enriched protein formulations and essential amino acid supplementation may be considered when conventional dietary strategies fail to achieve adequate protein targets, particularly in individuals with reduced appetite or gastrointestinal intolerance [47,48].

Accordingly, therapeutic strategies aimed at weight reduction should carefully balance the achievement of metabolic benefits with the preservation of LBM. Recent reports have highlighted the potential risk of suboptimal protein and micronutrient intake among individuals treated with GLP-1 RAs and GIP/GLP-1 RAs, reinforcing the importance of proactive nutritional assessment throughout treatment [53]. Increasing evidence also indicates that the preservation of FFM should be considered a therapeutic objective alongside fat loss, as excessive reductions in LBM may negatively affect skeletal muscle function, bone health, physical resilience, and long-term metabolic outcomes. Consequently, the quality of weight loss, rather than its magnitude alone, is increasingly recognized as a key determinant of healthy aging and successful obesity management [54].

From a practical perspective, nutritional management should be systematically integrated into the care of older adults receiving IBTs. Baseline and longitudinal nutritional assessment should include the evaluation of dietary protein intake, changes in appetite and food consumption, gastrointestinal tolerability, and alterations in body composition. Moderate hypocaloric dietary interventions should be individualized according to age, comorbidities, baseline nutritional status, functional reserve, and the magnitude of weight loss, while ensuring adequate protein and micronutrient intake. When dietary intake remains insufficient despite nutritional counseling, oral nutritional supplements, protein-enriched formulations, or targeted essential amino acid supplementation may be considered to help achieve recommended protein targets [47,48,50]. The assessment and correction of vitamin D deficiency, together with adequate calcium and micronutrient intake, should also be incorporated into routine clinical management because of their recognized role in maintaining musculoskeletal health [50].

Nutritional interventions should therefore be considered as part of a broader multimodal strategy aimed at preserving skeletal muscle health during IBT. In frail older adults or individuals with limited physiological reserve, the primary therapeutic objective should extend beyond maximizing weight loss toward optimizing body composition while preserving muscle strength, physical performance, and long-term functional independence [47,48,50,51,52,53,54]. The integration of nutritional optimization with structured physical activity represents the most comprehensive approach to maintaining muscle quantity and function during pharmacologically induced weight reduction.

#### 3.4.2. Exercise Interventions

Physical activity represents a pillar in the prevention and management of sarcopenia, particularly in older adults with diabesity. In the context of pharmacologically induced weight loss with agents such as semaglutide and tirzepatide, exercise assumes an even more critical role, as it may counterbalance reductions in LBM and preserve muscle function.

Aging is associated with progressive declines in muscle mass, strength, and neuromuscular function, which are further exacerbated by physical inactivity. In individuals with diabesity, these alterations are compounded by chronic low-grade inflammation, insulin resistance, and impaired muscle protein turnover. Weight loss interventions, although beneficial for metabolic health, may accelerate the loss of LBM if not accompanied by appropriate exercise strategies [8,49].

Among the different exercise modalities, resistance training is widely recognized as the most effective intervention for preserving and increasing SMM and strength in older adults [55,56]. Resistance exercise stimulates muscle protein synthesis, enhances neuromuscular recruitment, and improves muscle quality. Importantly, it may partially overcome anabolic resistance. In patients undergoing treatment with IBTs, resistance training can mitigate the LBM loss typically associated with a caloric deficit and pharmacologically induced weight reduction [57].

Aerobic exercise also contributes to metabolic and cardiovascular health, improving insulin sensitivity, mitochondrial function, and aerobic capacity, although its direct effect on muscle hypertrophy is less pronounced than that of resistance training. Therefore, combined programs incorporating both aerobic and resistance components are recommended to achieve comprehensive benefits [58,59].

Exercise may also influence qualitative aspects of skeletal muscle, such as mitochondrial density, capillary perfusion, and oxidative capacity, which are particularly relevant in patients treated with semaglutide or tirzepatide. Emerging evidence suggests that these therapies may improve aspects of muscle metabolic efficiency despite reductions in LBM, and exercise may synergize with pharmacological interventions to optimize functional outcomes [59,60].

Clinical guidelines recommend at least 2–3 weekly sessions of resistance training targeting major muscle groups, combined with ≥150 min/week of moderate-intensity aerobic activity, with intensity and volume tailored to baseline functional status and comorbidities. For frail or mobility-limited individuals, low-intensity or chair-based exercises provide meaningful benefits and can be progressively adapted according to individual capacity [8,61].

Adherence remains a major challenge in older adults. Supervised programs, social support, and integration into daily activities may improve compliance and long-term sustainability [58,60]. This is particularly important when rapid weight loss induced by semaglutide or tirzepatide may otherwise result in a disproportionate LBM loss. Regular exercise provides an anabolic stimulus, helping to maintain muscle strength, gait speed, and overall physical performance, which are critical determinants of independence and quality of life [53].

**Figure 2:**
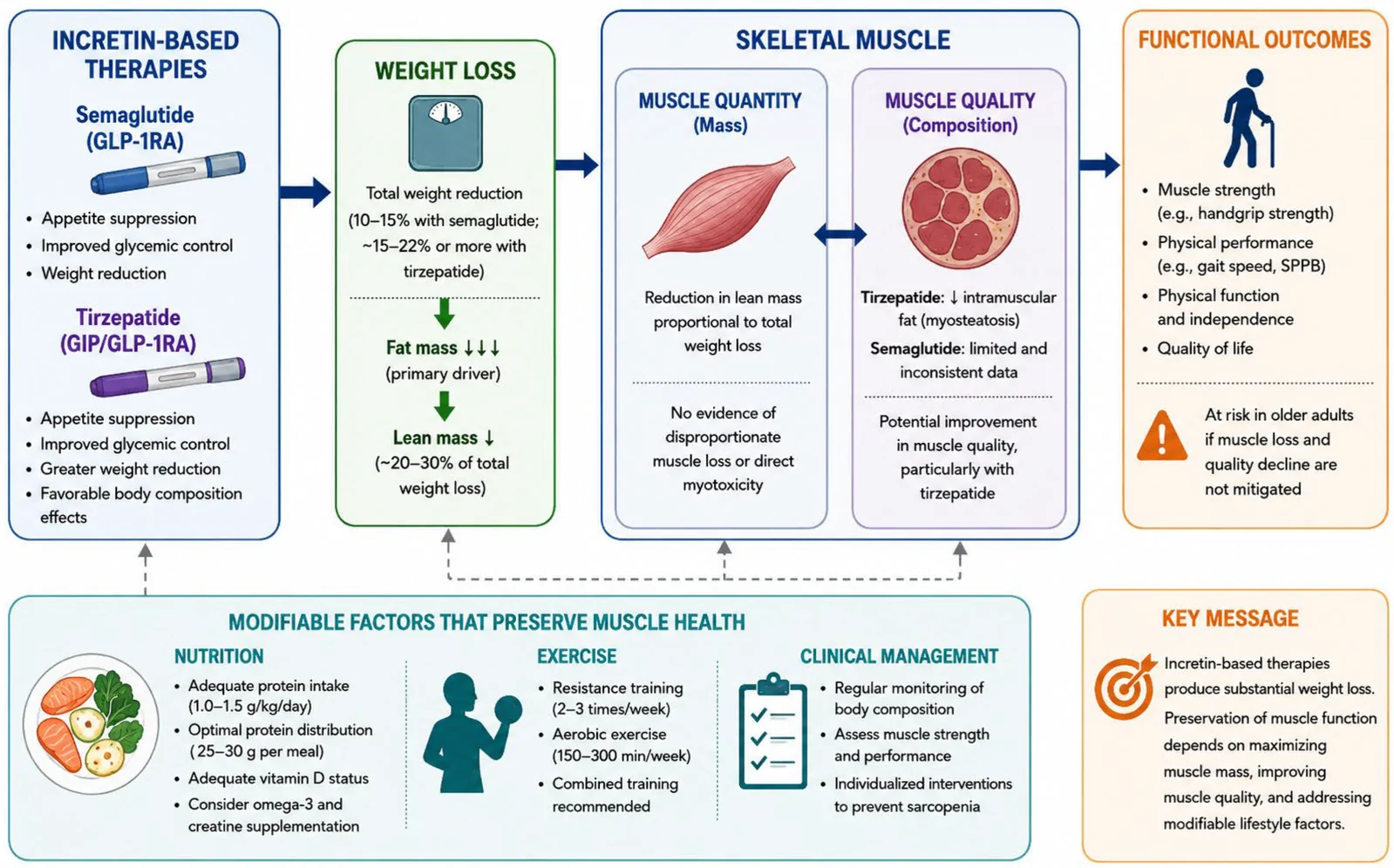
Conceptual model: interplay between IBTs, weight loss, muscle quantity, muscle quality and functional outcomes in older adults. **Abbreviations:** GIP, Glucose-dependent Insulinotropic Polypeptide; GLP1-RA, Glucagon-Like Peptide-1 Receptor Agonist; GLP1/GIP-RA, dual GLP-1/Glucose-dependent Insulinotropic Polypeptide Receptor Agonist; IBT, Incretin-Based Therapy; SPPB, Short Physical Performance Battery.

To offer a clinically grounded and comprehensive synthesis of the interplay between IBTs and lifestyle interventions, Figure 2 presents an integrated conceptual framework connecting pharmacologically driven weight loss with nutritional and exercise-related factors influencing SMM, muscle quality, and functional performance in older adults.

#### 3.4.3. Clinical Monitoring and Follow-Up Strategies

Beyond nutritional and exercise interventions, structured clinical monitoring is essential to identify patients at risk of clinically relevant muscle loss during IBT and to guide timely intervention. A pragmatic, stepwise monitoring approach is proposed below, acknowledging that no formally validated monitoring algorithm currently exists specifically for this population [8,42].

Baseline assessment, prior to treatment initiation, should include: (i) muscle strength, assessed by handgrip dynamometry; (ii) body composition, assessed by DXA where available or BIA as a more accessible alternative; (iii) physical performance, using gait speed or the SPPB, particularly in patients aged ≥65 years or with a clinical suspicion of sarcopenia; and (iv) a structured nutritional assessment of habitual protein intake and appetite.

Longitudinal monitoring should be individualized according to baseline risk, but a reasonable framework is to reassess body weight and handgrip strength at each routine visit, and to repeat body composition assessment (DXA or BIA) and formal physical performance testing at approximately 3, 6, and 12 months, or after each 5–10% increment of total body weight loss, as higher dosing and greater weight loss have been associated with an amplified functional decline in some cohorts [12,34].

Specific warning signs that should prompt closer evaluation, nutritional intensification, or a reconsideration of the treatment plan include: a decline in handgrip strength or gait speed disproportionate to the degree of weight loss, evidence of accelerated LBM loss compared with expected physiological ranges, unintentional weight loss exceeding therapeutic targets, or the emergence of new mobility limitations. In such circumstances, referral to a dietitian and/or physiotherapist, or evaluation by a geriatrician in older or frail patients, should be considered [47,48,56].

Given the current lack of dedicated trials, these monitoring strategies should ideally be prioritized in patients at the greatest risk of sarcopenia: those aged ≥65 years, individuals with pre-existing low muscle mass or strength, frail patients, and those achieving rapid or exceptionally large weight loss. A multidisciplinary approach, integrating endocrinology, clinical nutrition, and physical medicine, represents the most comprehensive strategy to safely preserve skeletal muscle health throughout IBT [8,56].

## 4. Limitations

The findings summarized in this narrative review should be interpreted in the context of several methodological limitations affecting both the available literature and the review design.

First, most studies investigating the effects of semaglutide and tirzepatide on skeletal muscle were not primarily designed to evaluate musculoskeletal outcomes. In many RCTs, body composition analyses were performed as post hoc analyses or imaging substudies, whereas skeletal muscle parameters represented secondary or exploratory endpoints. Consequently, much of the available evidence should be considered hypothesis-generating rather than definitive.

Second, considerable heterogeneity exists across studies regarding patient populations, treatment duration, methods used to assess skeletal muscle, and functional outcomes. Muscle quantity and quality were evaluated using different techniques, including DXA, BIA and MRI, while handgrip strength, gait speed, and physical performance were assessed inconsistently. This methodological heterogeneity limits direct comparisons across studies and complicates the interpretation of the clinical relevance of changes in muscle-related outcomes.

Furthermore, a substantial proportion of the available evidence derives from observational studies, real-world cohorts, relatively small prospective investigations, or post hoc analyses of larger RCTs. These study designs are inherently susceptible to selection bias, residual confounding, and limited external validity. Importantly, older adults, frail individuals, and patients with established sarcopenia remain underrepresented in the current literature, despite representing the population in whom the preservation of skeletal muscle is likely to be of the greatest clinical relevance.

Finally, this work was conducted as a narrative review and therefore does not follow the methodological framework of a systematic review. Although a comprehensive literature search was performed to identify the most relevant and recent evidence, no formal risk-of-bias assessment or quantitative synthesis was undertaken. Consequently, the conclusions should be interpreted as an integrated critical overview of the currently available evidence rather than as a formal evidence synthesis.

## 5. Conclusions

In summary, IBTs, including semaglutide and tirzepatide, induce substantial and clinically meaningful weight loss in individuals with diabesity while providing well-established metabolic and cardiometabolic benefits. However, their effects on skeletal muscle remain complex and are not yet fully characterized, particularly in older adults who are at an increased risk of sarcopenia, frailty, and functional decline.

Current evidence indicates that weight loss with GLP-1 RAs and GIP/GLP-1 RAs is accompanied by reductions in LBM. However, DXA- or BIA-derived LBM should not be interpreted as a direct surrogate for contractile skeletal muscle, and its reduction does not necessarily reflect equivalent muscle tissue loss or functional deterioration. Overall, LBM decline appears generally proportional to the magnitude of weight loss and consistent with physiological adaptations to energy restriction, although interpretation remains limited by heterogeneity in populations, assessment methods, and functional outcomes.

Beyond muscle quantity, emerging evidence highlights muscle quality as an important determinant of skeletal muscle health. Tirzepatide appears to induce favorable changes in body composition, including reductions in MFI, suggesting potential improvements in muscle quality despite reductions in muscle volume. Whether these changes translate into meaningful improvements in strength or physical performance, particularly in older and frail individuals, remains unknown. Findings regarding semaglutide are more heterogeneous, with variable effects on muscle strength and functional outcomes. Thus, the clinical relevance of lean tissue changes likely depends not only on the magnitude of LBM reduction but also on baseline muscle reserve, nutritional status, physical activity, and overall vulnerability.

Available evidence does not support a direct myotoxic effect of IBTs. Rather, skeletal muscle changes appear to be primarily driven by the energy deficit associated with weight loss and modulated by individual factors, including age, disease burden, baseline muscle quality, nutritional adequacy, and physical activity level. Nonetheless, clinically relevant functional decline may occur in susceptible individuals, emphasizing the need for careful longitudinal monitoring, particularly in older adults and those with limited physiological reserve.

From a clinical perspective, these findings support moving beyond a weight-centric approach and considering the preservation of skeletal muscle as an integral component of successful IBT. Optimizing outcomes requires adequate energy and high-quality protein intake, individualized nutritional assessment, resistance exercise, and regular evaluation of muscle strength and physical performance, particularly in patients with or at risk of sarcopenic obesity. In this context, the quality of weight loss should be regarded as equal in importance to its magnitude, with the preservation of FFM and functional capacity representing key therapeutic objectives.

Future research should prioritize standardized and multidimensional approaches to skeletal muscle assessment, integrating measures of LBM, muscle strength, physical performance, and imaging-based indicators of muscle quality. Dedicated studies in older and frail individuals are urgently needed, together with trials evaluating whether targeted nutritional interventions, including the optimization of protein intake and selected nutritional supplementation, can further preserve skeletal muscle health during IBT.

Ultimately, the success of IBT should not be defined solely by the amount of weight lost, but by the ability to achieve a favorable body composition while preserving skeletal muscle quality, physical performance, metabolic health, and long-term functional independence. Particularly in older adults, the therapeutic goal should extend beyond weight reduction toward promoting healthy aging through an integrated, multidisciplinary approach.

## Figures and Tables

**Table 1:** Clinical and methodological characteristics of studies evaluating incretin-based therapies and skeletal muscle outcomes.

Study (Ref)	Design	Population (*N*; Age/Setting)	Intervention and Dose	Duration	Assessment Methods	Key Skeletal-Muscle Findings
STEP 1 (Wilding et al.) [5]	RCT	*N* = 1961 (semaglutide *n* = 1306; placebo *n* = 655); adults with obesity/overweight, no T2DM	Semaglutide 2.4 mg/week	68 wk	Body weight, cardiometabolic endpoints (no dedicated DXA substudy)	Weight loss 14.9% vs. 2.4%; body composition substudies of the wider STEP program show proportional LBM reduction
Volpe et al. [10]	Prospective real-life study	Adults with T2DM	Oral semaglutide	26 weeks	BIA	Improved body composition with preservation of LBM
SEMALEAN (Alissou et al.) [11]	Prospective real-world cohort	*N* = 115 enrolled/106 completed; adults with obesity	Semaglutide 2.4 mg/week	12 months (M0, M7, M12)	DXA, handgrip strength, resting energy expenditure	LBM declined then stabilized; handgrip strength increased; sarcopenic obesity prevalence 49%→33%
Ren et al. [12]	Retrospective cohort	*N* = 220 semaglutide vs. 212 controls; older adults (≥65 y) with T2DM	Semaglutide	24 months	Appendicular skeletal muscle index, handgrip strength, gait speed	Accelerated decline in SMM and physical performance; dose and baseline muscle index were predictors
SUSTAIN 8 DXA substudy (McCrimmon et al.) [13]	RCT substudy	*N* = 178; adults with T2DM	Semaglutide 1.0 mg/week vs. canagliflozin 300 mg/day	52 wk	DXA	Reductions in both FM and LBM with semaglutide
Ozeki et al. [14]	Prospective pilot study	Small pilot cohort; elderly patients with obesity and T2DM	Semaglutide	3 months	BIA	Reduced FM and SMM; preserved relative muscle percentage and limb distribution
Qaisar et al. [15]	Prospective observational	N = 141 (semaglutide n = 68; sitagliptin *n* = 73); older men with T2DM	Semaglutide vs. sitagliptin	12 months	Handgrip strength, gait speed, muscle index, SPPB, NMJ biomarkers	Declines in strength and physical performance; increased biomarkers of NMJ degradation
Pantanetti et al. [16]	Real-world cohort	Adults with T2DM	Semaglutide (subcutaneous)	Real-world follow-up	BIA	Reduction in LBM despite metabolic improvement
SLIM LIVER (Ditzenberger et al.) [17]	Prospective cohort	Adults	Semaglutide	Not specified	Imaging (psoas muscle volume), physical performance testing	Reduced psoas muscle volume without significant deterioration in physical performance
Cortes et al. [18]	RCT protocol (ongoing)	Older adults with overweight and insulin resistance	Semaglutide	Ongoing	Muscle strength, physical performance, body composition, aging biomarkers	Results pending; designed to clarify functional relevance of body composition changes
SURMOUNT-1 DXA substudy (Villareal et al.) [19]	RCT post hoc substudy	*N* = 255; adults with obesity/overweight, no T2DM	Tirzepatide 15 mg/week	72 wk	DXA	Weight −21.4%; FM −25.7% vs. placebo; LBM −8.3%; ~74% of total loss from FM
SURPASS-3 MRI substudy (Sattar et al.) [20]	RCT post hoc substudy	*N* = 246 (pooled tirzepatide n = 190; insulin degludec *n* = 56); adults with T2DM, mean age 56 y	Tirzepatide 5/10/15 mg/week vs. insulin degludec	52 wk	MRI (thigh muscle volume, muscle fat infiltration)	Muscle volume decline proportional to weight loss vs. UK Biobank estimates; MFI decreased more than predicted
Hidalgo Ramos et al. [21]	Systematic review	Multiple RCTs (SURMOUNT-1, SURPASS-3 substudies); adults	Tirzepatide	Variable (per included trial)	DXA/MRI evidence synthesis	~75% of weight loss attributable to FM; LBM reduction proportional; improved MFI

**Abbreviations:** BIA, Bioelectrical Impedance Analysis; DXA, Dual-Energy X-ray Absorptiometry; MRI, Magnetic Resonance Imaging; RCTs, Randomized Controlled Trials; T2DM, Type 2 Diabetes Mellitus; MFI, Muscle Fat Infiltration; FM, Fat Mass; LBM, Lean Body Mass, SMM, Skeletal Muscle Mass; SPPB, Short Physical Performance Battery; NMJ, NeuroMuscular Junction.

**Table 2:** Comparative effects of semaglutide and tirzepatide on skeletal muscle: an integrated evidence synthesis.

Domain	Semaglutide	Tirzepatide	Clinical Interpretation	Key Refs
**Weight loss magnitude**	High (≈10–15%)	Very high (≈15–22% or more)	Greater total energy deficit with tirzepatide	[5,6,19,34]
**FM reduction**	Predominant	More pronounced	Primary driver of body weight reduction for both agents	[6,19,20]
**LBM changes**	Moderate reduction proportional to weight loss	Similar proportional reduction (~20–30% of weight loss)	No evidence of disproportionate skeletal muscle catabolism	[13,19,20]
**Muscle strength**	Generally preserved; variable findings in older or frail adults	Limited data; generally preserved in clinical trials	Functional outcomes appear more vulnerable than muscle mass in high-risk populations	[12,15,35]
**Muscle quality**	Limited evidence; heterogeneous findings	Improved muscle composition (↓ MFI on MRI)	Potential qualitative advantage of tirzepatide despite LBM reduction	[20,43,44]
**Mechanism of muscle change**	Energy deficit, reduced caloric intake, improved metabolic control	Energy deficit combined with GIP/GLP-1-mediated effects on nutrient partitioning	No evidence of direct myotoxicity for either agent	[46]
**Older adults**	Heterogeneous outcomes; possible decline in muscle strength and physical performance in frail individuals	Insufficient direct evidence; possible benefit on muscle quality remains speculative	Older adults represent the major evidence gap	[12,15,18]
**Evidence quality**	Moderate; based on RCTs, observational studies and real-world evidence	Moderate; mainly based on DXA and MRI substudies of RCTs	Functional outcomes remain insufficiently investigated	[see Table 3]
**Clinical priority**	Preserve muscle function during weight loss	Optimize body composition while preserving muscle quality	Combine pharmacotherapy with adequate protein intake, resistance exercise, and longitudinal clinical monitoring	[47,48,49]

**Abbreviations:** GIP, Glucose-dependent Insulinotropic Polypeptide; GLP-1RA, Glucagon-Like Peptide-1 Receptor Agonist; LBM, Lean Body Mass; MFI, Muscle Fat Infiltration; MRI, Magnetic Resonance Imaging; DXA, Dual-energy X-ray Absorptiometry; RCTs, Randomized Controlled Trials.

**Table 3:** Quality assessment of the evidence addressed in this narrative review, organized by evidence stream.

Evidence Stream	Study Design	Approx. No. of Sources	Main Risk-of-Bias Concerns	Overall Quality *
RCT-derived DXA/body composition substudies (STEP, SURMOUNT, SURPASS, SUSTAIN programs) [5,6,13,19,20]	RCTs/post hoc substudies	~6	Body composition mostly a secondary/exploratory endpoint; selective substudy populations	Moderate
Prospective real-world cohorts (SEMALEAN, Volpe et al., Ozeki et al.) [10,11,14]	Prospective observational	3	No control group in most; limited representation of older/frail adults	Low-to-moderate
Retrospective/observational studies focused on older adults (Ren et al., Qaisar et al.) [12,15]	Retrospective/observational	2	Residual confounding; relatively small samples; single-sex cohort (Qaisar et al.)	Low
Real-world bioimpedance-based cohorts (Pantanetti et al.) [16]	Real-world cohort	1	No control group; BIA less precise than DXA/MRI	Low
Imaging substudies (SLIM LIVER, SURPASS-3 MRI) [17,20]	Post hoc imaging substudy	2	Small imaging subsamples; comparison with external population cohorts (e.g., UK Biobank) is indirect	Moderate
Systematic review/evidence synthesis (Hidalgo Ramos et al.) [21]	Systematic review	1	Included studies are heterogeneous; no formal meta-analysis performed	Moderate
Ongoing/protocol studies (Cortes et al.) [18]	RCT protocol (not yet completed)	1	Results not yet available	Not yet assessable

* Quality ratings are qualitative judgments reflecting study design, risk of bias, and consistency of findings across the narrative literature search described in Section 2; they do not reflect a formal GRADE or Cochrane risk-of-bias assessment, consistent with the narrative (non-systematic) design of this review (see Section 4, Limitations). **Abbreviations:** BIA, Bioelectrical Impedance Analysis; DXA, Dual-Energy X-ray Absorptiometry; MRI, Magnetic Resonance Imaging; RCTs, Randomized Controlled Trials.

## Data Availability

No new data were created or analyzed in this study. Data sharing is not applicable to this article.

## Abbreviations

BIA, Bioelectrical Impedance Analysis; CKD, Chronic Kidney Disease; DXA, Dual-Energy X-Ray Absorptiometry; EWGSOP2, European Working Group on Sarcopenia in Older People; FM, Fat Mass; FFM, Fat-Free Mass; FNIH, Foundation for the National Institutes of Health; GIP, Glucose-dependent Insulinotropic Polypeptide; GLP1-RA, Glucagon-Like Peptide-1 Receptor Agonist; GLP-1/GIP-RA, dual Glucagon-Like Peptide-1/Glucose-dependent Insulinotropic Polypeptide Receptor Agonist; HMB, β-Hydroxy-β-Methylbutyrate; IBT, Incretin-Based Therapy; LBM, Lean Body Mass; MFI, Muscle Fat Infiltration; MRI, Magnetic Resonance Imaging; NMJ, Neuromuscular Junction; ω-3, Omega-3; PUFA, Polyunsaturated Fatty Acid; RCT, Randomized Controlled Trial; SDOC, Sarcopenia Definition and Outcomes Consortium; SGLT2i, Sodium-Glucose Co-Transporter-2 inhibitors; SMM, Skeletal Muscle Mass; SPPB, Short Physical Performance Battery; T2DM, Type 2 Diabetes Mellitus.
